# The Inlay Matrix

**Published:** 1907-10-15

**Authors:** J. Q. Byram


### THE INLAY MATRIX.

BY J. Q. BYRAM, D.D.S.

In constructing matrices we have three general methods Those of you in this section of the country, of course, are familiar with Dr. Peck's system where he constructs his matrix by swedging over an impression of the cavity. Then we have the method of burnishing directly into the cavity and the method of swaging into the model of the cavity. For all cavities on the labial or buccal surfaces of teeth I use the Peck method. That is, I take impressions of the cavities and swedge over the impression. But for simple approximal cavities and approximo-incisal cavities I prefer to burnish the matrix directly into the cavity. Those of us who have been doing inlay work for a considerable length of time have quite a collection of burnishers. I have discarded all but four ball burnishers, one or two blade burnishers, a pair of special burnishers for burnishing the matrix in the gingival region where the cavity extends to or beneath the gum margin and one burnisher for going over the axial margins.

In the early days of inlay work I spent quite a bit of time in trying to work matrices into cavities without having tears. After trying for a year or more without succeeding, I decided that tears made but little difference. So, unless it is a large tear at the margin I pay no attention to it. I spent a good deal of time using cotton and all such accessories trying to burnish the matrix into the cavity and at the present time let me say that I seldom use cotton or punk when I am using platinum. I use the 1-1000 platinum foil exclusively and I use the ball burnishers in most cases from the beginning. In using the ball burnishers I start with the largest size and in case the cavity extends beneath the gum margin I first fold the foil so that when it is worked down against the gingival margin there will be a surplus of material in that region. Otherwise it may draw in such manner that the margin will not be covered. The matrix may be worked down into the center of the cavity with ball burnishers taking the large burnishers first.

One point to which I wish to call your attention in cavities with one or more reverse curves, is that the foil should be left as nearly in a plane surface as it can except that portion in the cavity. If this were worked down in the cavity as you see outlined here, working it first with a large ball burnisher, continuing with the different sizes, it can be forced into the grooves with only a few small tears. After the matrix has been burnished with the ball burnishers, then I deem it advisable to use the method which is familiar to you all; that is the method of placing a small amount of camphor in the cavity. After the camphor has been packed into the cavity, I find that the best material to use in drawing the matrix to place is a piece of rubber dam. While I use tape a great deal, I have discarded its use at this stage of the operation, for one of the delicate points of the operation is placing the rubber over the matrix so that it swedges under direct pressure and does not tear the matrix at the margin. At this stage of the operation the foil should be trimmed to proper size. Notice, please, that at this time it should be worked down with ball burnishers so that it fits in the groove between the plates, using the large burnishers first and afterwards working it down with the smaller ones. If the cavity extend beneath the gingival margin, I find by taking the special gingival margin burnishers the foil can be carried beneath the gum margin so that it is not at all painful to the patient and at the same time it can be burnished in such manner that a good margin is obtained.

After I have the matrix well in shape, if it begins to work somewhat elastic, I anneal it; otherwise I do not. I used to spend a great deal of time annealing matrices. Usually I anneal once in these large cases.

At this point I use sticky wax by taking a piece of the ordinary crown and bridge wax (which is commonly known as sticky wax) and holding it near the flame until it becomes slightly plastic and then after immersing the ends of the fingers in water I take a small piece and press in the cavity. (It is more adhesive and just a little more rigid than camphor.)

After the wax has been pressed into the cavity, if the margins need burnishing I use the face of the burnisher over tape.

After that is done, I do the remainder of the burnishing by the principle of swedging. If necessary place a little more wax in the matrix and then I place the tape over and simply swedge the margins to place, probably swaging four or five times and using anywhere from twenty to thirty pounds pressure in swedging.

In case of small tears at the margin, instead of making a new matrix, I take a small amount of a thick solution of shellac and drop it on the cavity side of the matrix in the region of the tear. You will find it will bridge in the space and prevent the porcelain from running through the break. Our trouble with the matrix with the tear at the margin is not that it affects the margin but the small amount of porcelain that gets on the under side adheres to the foil and causes the margin of the inlay to chip in getting the platinum off. I advise you not to try using shellac on the margin with De Trey's porcelain. That method can be used with the Jenkins' porcelain provided the shellac is burned out slowly. I seldom use that method except when I use high fusing porcelain, and I might say that I seldom construct a large approximal-incisal inlay that I do not use high fusing porcelain.

For the benefit of those who swage matrices I have found plungers made of vellum rubber to be the most efficient, and I have found in all places where I swage into the model of the cavity, by using a plunger conical in shape the material is forced into the center of the cavity and spreads from the center to the margins so that there is less danger of tearing the matrix at the margin. If there is a tear it is in the center. Whereas if you take a material with a flat surface and swage in the same cavity you must take a great deal more time to work the matrix to place.—*Tri-State Record.*

